# Case Report on: Very Early Afterdepolarizations in HiPSC-Cardiomyocytes—An Artifact by Big Conductance Calcium Activated Potassium Current (I_bk,Ca_)

**DOI:** 10.3390/cells9010253

**Published:** 2020-01-20

**Authors:** András Horváth, Torsten Christ, Jussi T. Koivumäki, Maksymilian Prondzynski, Antonia T. L. Zech, Michael Spohn, Umber Saleem, Ingra Mannhardt, Bärbel Ulmer, Evaldas Girdauskas, Christian Meyer, Arne Hansen, Thomas Eschenhagen, Marc D. Lemoine

**Affiliations:** 1 Institute of Experimental Pharmacology and Toxicology, University Medical Center Hamburg-Eppendorf, 20246 Hamburg, Germany; bandi185@yahoo.com (A.H.); mprondzynski@gmail.com (M.P.); a.zech@uke.de (A.T.L.Z.); u.saleem@uke.de (U.S.); i.mannhardt@uke.de (I.M.); b.ulmer@uke.de (B.U.); ar.hansen@uke.de (A.H.); t.eschenhagen@uke.de (T.E.); 2 DZHK (German Center for Cardiovascular Research), Partner Site Hamburg/Kiel/Lübeck, 20246 Hamburg, Germany; e.girdauskas@uke.de; 3 Department of Pharmacology and Pharmacotherapy, Faculty of Medicine, University of Szeged, 6721 Szeged, Hungary; chr.meyer@uke.de; 4 BioMediTech, Faculty of Medicine and Health Technology, Tampere University, 33520 Tampere, Finland; j.koivumaeki@gmail.com; 5 Bioinformatics Core, University Medical Center Hamburg-Eppendorf, 20246 Hamburg, Germany; m.spohn@uke.de; 6 Department of Cardiovascular Surgery, University Heart Center, 20246 Hamburg, Germany; 7 Department of Cardiology-Electrophysiology, University Heart Center, 20246 Hamburg, Germany

**Keywords:** human induced pluripotent stem cell-derived cardiomyocytes (hiPSC-CMs), iPS cells, stem cells, big conductance calcium activated potassium channel (BK), Maxi-K, slo1, K_Ca1.1_, iberiotoxin, long QT syndrome

## Abstract

Human induced pluripotent stem cell-derived cardiomyocytes (hiPSC-CMs) represent an unlimited source of human CMs that could be a standard tool in drug research. However, there is concern whether hiPSC-CMs express all cardiac ion channels at physiological level and whether they might express non-cardiac ion channels. In a control hiPSC line, we found large, “noisy” outward K^+^ currents, when we measured outward potassium currents in isolated hiPSC-CMs. Currents were sensitive to iberiotoxin, the selective blocker of big conductance Ca^2+^-activated K^+^ current (I_BK,Ca_). Seven of 16 individual differentiation batches showed a strong initial repolarization in the action potentials (AP) recorded from engineered heart tissue (EHT) followed by very early afterdepolarizations, sometimes even with consecutive oscillations. Iberiotoxin stopped oscillations and normalized AP shape, but had no effect in other EHTs without oscillations or in human left ventricular tissue (LV). Expression levels of the alpha-subunit (K_Ca_1.1) of the BK_Ca_ correlated with the presence of oscillations in hiPSC-CMs and was not detectable in LV. Taken together, individual batches of hiPSC-CMs can express sarcolemmal ion channels that are otherwise not found in the human heart, resulting in oscillating afterdepolarizations in the AP. HiPSC-CMs should be screened for expression of non-cardiac ion channels before being applied to drug research.

## 1. Introduction

Human induced pluripotent stem-cell derived cardiomyocytes (hiPSC-CMs) have gained interest as a human model to study heart physiology and pathophysiology [1,2,3,4], cardiovascular pharmacology [5] and cardiac repair [6]. In this context, it is important that hiPSC-CMs share properties of human adult CMs. Many reports claimed that characteristics of hiPSC-CMs might differ from adult CMs, a finding frequently interpreted as immaturity. On the one hand, immaturity could be caused by the lack of cardiac ion channels or by differences in expression levels such as the inward rectifier potassium current (I_K1_) [7,8,9,10]. On the other hand, hiPSC-CMs can show ion currents, which are absent in adult human CMs such as the T-type calcium current [11,12]. Based on these findings, there is a need for a detailed electrophysiological characterization of hiPSC-CMs before using them as a model for human CMs. Here we report the coincidently found expression of a non-cardiac ion channel in hiPSC-CMs as a peculiarity of a single control cell line: the big conductance calcium activated potassium current (BK_Ca_; alternatively used names: Maxi-K, slo1, K_Ca1.1_).

BK_Ca_ is a voltage- and calcium-gated potassium channel (K_Ca1.1_) generating huge conductivity for potassium. BK_Ca_ is widely expressed in the human body, mainly in neural cells, blood vessels, kidney, but not in in cardiomyocytes. Recently, artificial expression of BK_Ca_ was shown to be able to shorten action potentials (APs) in murine CMs [13]. Consequently, expression of the non-cardiac BK_Ca_ current in human CMs was proposed as a genetic therapy for the long QT syndrome. However, the contribution of individual ion channels to repolarization and resulting AP shape differs from murine to human CMs. Putative impact of BK_Ca_ to human cardiac electrophysiology remains unclear.

The goal of this study was to draw attention to the fact that hiPSC-CMs can express non-cardiac ion channels. In addition, we had the chance to elucidate how the expression of BK_Ca_ may influence the membrane potential of human CMs. Based on our findings, we propose a regular assessment for expression of non-cardiac ion channels as a part of quality control when using hiPSC-CMs.

## 2. Methods

### 2.1. Generation of hiPSC and Engineered Heart Tissue (EHT)

The hiPSC line C25 (kind gift from Alessandra Moretti, Munich, Germany) was reprogrammed by lentivirus [1] and was expanded in FTDA medium and differentiated in a three step protocol based on growth factors and a small molecule Wnt inhibitor DS07 (kind gift from Dennis Schade, Dortmund, Germany) as previously published [14,15,16]. The hiPSC line ERC018 were generated in-house from skin fibroblasts of a healthy subject using the CytoTune^TM^-iPS Sendai Reprogramming Kit (Thermo Fisher Scientific, Waltham, MA, USA) and differentiated to cardiomyocytes as described for C25. In brief, confluent undifferentiated cells were dissociated (0.5 mM EDTA; 10 min) and cultivated in spinner flasks (30 × 10^6^ cells/100 mL; 40 rpm) for embryoid body formation overnight. Mesodermal differentiation was initiated in embryoid bodies over three days in suspension culture with growth factors (BMP-4 (R&D Systems, 314-BP), activin-A (R&D Systems, 338-AC) and FGF2 (PeproTech, 100-18B)). Cardiac differentiation was performed either in adhesion or in suspension culture with Wnt-inhibitor DS07. Cells were cultured in a humidified temperature and gas-controlled incubator (37 °C, 5% CO_2_, 5% O_2_ and 21% O_2_ for final cardiac differentiation). At day 14 the spontaneously beating hiPSC-CMs were dissociated with collagenase II (Worthington, LS004176; 200 U/mL, 3.5 h). For quality control, dissociated cells were analyzed by flow cytometry as described before [14,17] with anti-cardiac troponin T-FITC (Miltenyi, clone REA400, 130-112-756). All differentiation runs utilized for this study had 64–96% cardiac Troponin T positive cells (Supplementary Materials Figure S2). Further characterization of the non-CMs within the EHT was evaluated previously by our group [6], showing low expression of vimentin-positive fibroblast-like markers and the virtual absence of endothelial, neuronal, and endodermal markers. ICell and iCell² cardiomyocytes are commercially available hiPSC-CM lines purchased from Fujifilm Cellular Dynamics (Madison, Wisconsin, USA) and were included in expression analysis after culture in EHT. For three-dimensional culture EHTs were generated with 1 × 10^6^ hiPSC-CM/100 µL EHT mastermix as previously described [18]. EHTs were cultured in a 37 °C, 7% CO_2_ and 40% O_2_ humidified cell culture incubator with a medium consisting of DMEM (F0415, Biochrom; Berlin, Germany), 10% heat-inactivated horse serum (Gibco 26050, Thermo Fisher Scientific, Waltham, MA, USA), 1% penicillin/streptomycin (Gibco 15140), insulin (10 µg/mL; Sigma-Aldrich I9278, St. Louis, MO, USA) and aprotinin (33 µg/mL; Sigma-Aldrich A1153). EHTs were cultured for at least 3 weeks to allow maturation. The work with hiPSC was approved by the Ethical Committee of the University Medical Center Hamburg-Eppendorf (Az. PV4798, 28.10.2014). All donors gave written informed consent.

### 2.2. Human Adult Heart Tissue

This investigation conforms to all principles outlined by the Declaration of Helsinki and the Medical Association of Hamburg. All materials from patients were taken with informed consent of the donors. Left ventricular free wall and left ventricular septum samples were obtained from patients undergoing heart transplantation or from aortic valve surgery.

### 2.3. Current Recordings

HiPSC-CMs in EHT were isolated with collagenase II for 5 h (200 U/mL, Worthington, LS004176 dissolved in HBSS-Puffer without Mg^2+^ or Ca^2+^, Gibco 14175-053 and 1 mM HEPES; pH 7,4), and re-plated on gelatin-coated coverslips for 24–48 h in order to maintain adherence under perfusion. Outward K^+^ currents were measured at 37 °C, using the whole-cell configuration of the patch clamp technique. Axopatch 200B amplifier (Axon Instruments, Foster City, CA, USA) and ISO2 software were used for data acquisition and analysis (MFK, Niedernhausen, Germany). Heat-polished pipettes were pulled from borosilicate filamented glass (Hilgenberg, Malsfeld, Germany). Tip resistances were 2.5–5 MΩ when filled with pipette solution. Seal resistances were 2–4 GΩ. The cells were investigated in a small perfusion chamber placed on the stage of an inverse microscope. Application of drugs was performed by a system for rapid solution changes (Cell Micro Controls, Virginia Beach, VA, USA; ALA Scientific Instruments, Long Island, NY, USA) [19]. The experiments were performed with the following bath solution (in mM): NaCl 120, KCl 5.4, HEPES 10, CaCl_2_ 2, MgCl_2_ 1 and glucose 10 (pH 7.4, adjusted with NaOH). Outward currents were elicited by 1000 ms depolarizing test pulses from −80 to +70 mV (0.2 Hz). The pipette solution included (in mM): DL-Aspartate potassium salt 80, KCl 40, NaCl 8, HEPES 10, Mg-ATP 5, Tris-GTP 0.1, EGTA 5 and CaCl_2_ 4.4, pH 7.4, adjusted with KOH [20].

### 2.4. AP Recordings

To record APs in intact EHT and in left ventricular trabeculae we used sharp microelectrodes as reported previously [5,9,21]. Microelectrode tip resistances were 20–50 MΩ when filled with 3 mM KCl. APs were elicited by field stimulation at 1 Hz, 0.5 ms stimulus duration and 50% above threshold intensity. The following bath solution was used (in mM): NaCl 125, KCl 5.4, MgCl_2_ 0.6, CaCl_2_ 1, NaH_2_PO_4_ 0.4, NaH_2_CO_3_ 22 and glucose 5.5 and was equilibrated with O_2_–CO_2_ (95:5). The experiments were performed at 37 °C.

### 2.5. Molecular Biology

Total RNA was extracted from snap frozen LV and EHT using an RNAeasy mini Kit (Qiagen, Valencia, CA, USA). RNA concentration was determined per fluorometric quantitation with Qubit^TM^ (Thermo Fisher Scientific; Waltham, MA, USA) according to the manufacturer’s instructions. Of total RNA 50 ng was used for expression analysis by nanoString nCounter^®^ SPRINT Profiler according to the manufacturer’s instructions. Raw data were analyzed with nSolver^TM^ Data Analysis Software including background subtraction using negative controls and normalization to two housekeeping genes (GAPDH and PGK1).

### 2.6. Mathematical Modelling and Computer Simulations

To simulate the BK_Ca_ current in silico, we extended a previously published formulation [22] to include (1) calcium dependence according to the Lin et al. [23] data, and (2) inactivation kinetics according to Ding et al. [24] data. To demonstrate the contribution of BK_Ca_ current on AP repolarization, we integrated the BK_Ca_ model to a well-established human left ventricular CM model [25].

### 2.7. Statistical Analysis

Results are presented as mean values ± SEM. Area under the curve was calculated by using the GraphPad Prism Software 5.02 (GraphPad Software, San Diego, CA, USA). Statistical differences were evaluated by using the Student’s *t*-test (paired or unpaired) or repeated measures ANOVA, followed by a Bonferroni test, where appropriate. A value of *p* < 0.05 was considered to be statistically significant.

### 2.8. Drugs

All drugs and chemicals were obtained from Sigma-Aldrich (St. Louis, MI, USA) except for iberiotoxin (IBTX, Tocris Bioscience, Bristol, UK).

## 3. Results

### 3.1. Outward Potassium Currents in hiPSC-CMs, Appearance of I_BK,Ca_

**Figure 1:**
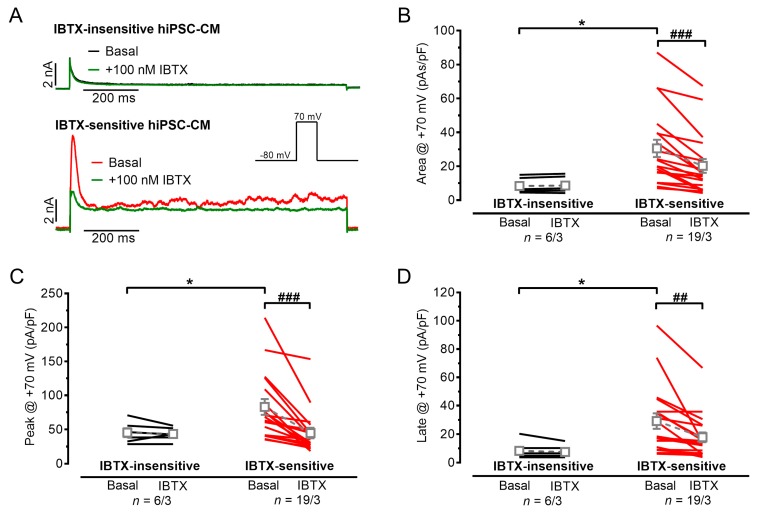
Outward currents in C25 human induced pluripotent stem cell-derived cardiomyocytes (hiPSC-CMs) and the effect of iberiotoxin (IBTX). (**A**) Original outward traces before (black) and after (green) exposure of 100 nM IBTX in insensitive (upper, black directly underlying green curve) and sensitive (lower panel) hiPSC-CMs. (**B**–**D**). Summary of IBTX (100 nM) effects in insensitive (left panel) and sensitive (right panel) hiPSC-CMs quantified by area under the curve (**B**), peak current (**C**) and current at the end of the test pulse (late current, **D**). Mean values ± SEM. * *p* < 0.05, unpaired Student’s *t* test for basal values in insensitive vs. sensitive hiPSC-CMs; ^##^ *p* < 0.01, ^###^ *p* < 0.001; paired Student’s *t* test for basal vs. IBTX; *n* = number of isolated cells/number of individual differentiation batches.

Large, transient outward currents were elicited in hiPSC-CMs (Figure 1A) by depolarizing test pulses. We found in several C25-hiPSC-CMs a large, inactivating outward current followed by a late sustained current with an irregular shape during the entire depolarizing test pulse. The irregular-shaped “noisy” currents were similar to BK_Ca_ currents, which were reported previously in mesenchymal stem cells [26]. Similar to that report we used rather high test pulse potentials (+70 mV) from physiological resting membrane potential of −80 mV and increased the free Ca^2+^ concentration of the pipette solution from 2 to 4.4 mM to facilitate the detection of BK_Ca_ [26]. The selective I_BK,Ca_ blocker IBTX (100 nM) was used to identify the I_BK,Ca_ (Figure 1A). Of the hiPSC-CMs 76% (19 out of 25) showed IBTX-sensitive outward currents suggesting the presence of BK_Ca_; the area under the curve was reduced by IBTX from 30.6 ± 5.1 pAs/pF to 20.2 ± 4.1 pAs/pF (*n* = 19, *p* < 0.0001, paired *t* test, Figure 1B). IBTX inhibited both the peak and late current density (peak from 82.9 ± 11.5 pA/pF to 44.8 ± 7.6 pA/pF, *p* < 0.0001, Figure 1C and late: from 29.2 ± 5.4 pA/pF to 17.8 ± 3.4 pA/pF; *n* = 19, *p* = 0.004, Figure 1D). In IBTX-insensitive hiPSC-CMs, baseline values of outward peak and late currents were smaller compared to IBTX-sensitive hiPSC-CMs. Furthermore, in hiPSC-CMs without the irregular-shaped outward current IBTX did not change peak or late currents (peak: 45.4 ± 6.4 pA/pF baseline vs. 43.4 ± 3.9 pA/pF IBTX, *p* = 0.594, *n* = 6 and late: 8.3 ± 2.6 pA/pF baseline vs. 7.5 ± 1.8 pA/pF IBTX, *n* = 6, *p* = 0.363; paired *t* test). HiPS-CMs from the commercially available iCell cell line did not show irregular-shaped “noisy” currents or IBTX-sensitivity (Supplementary Materials Figure S1).

### 3.2. Action Potentials with Strong Initial Repolarization and Oscillations Are Sensitive to Iberiotoxin

**Figure 2:**
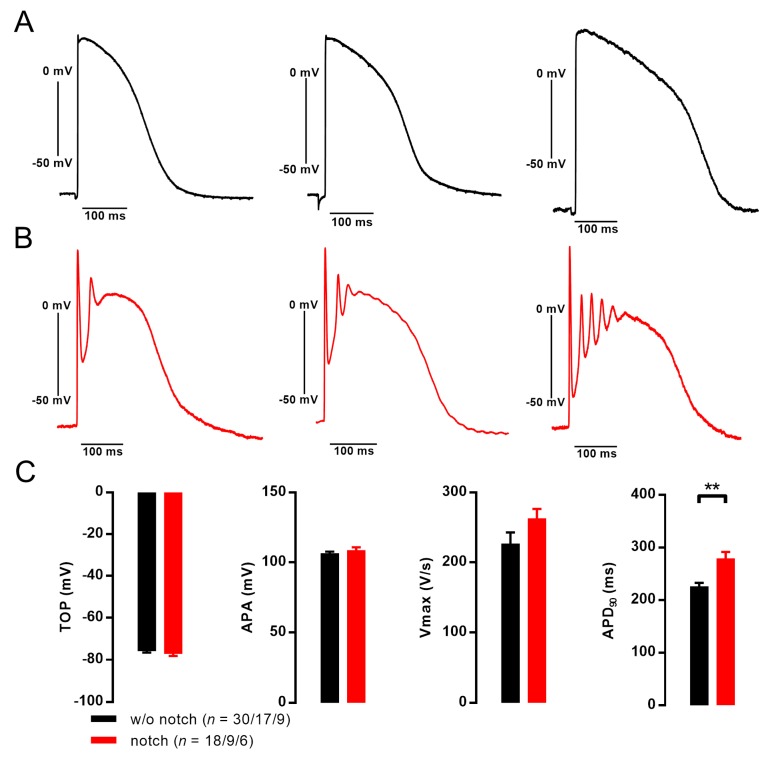
Pronounced notch with oscillation in the plateau phase of action potential (AP) recorded from engineered heart tissue (EHT) derived by C25 human induced pluripotent stem cell-derived cardiomyocytes (hiPSC-CMs). (**A**) Original AP recordings without (**A**) and with (**B**) notch followed by oscillating afterdepolarizations in EHT from cell line C25. (**C**) Summary of the results for take-off potential (TOP), AP amplitude (APA), maximum upstroke velocity (V_max_), AP duration at 90% repolarization (APD_90_), ** *p* < 0.01, unpaired Student’s *t* test; *n* = number of impalements/number of EHTs/number of individual differentiation batches.

APs recorded in C25-EHTs, exhibiting the IBTX-sensitive irregular-shaped outward current, showed a pronounced initial repolarization (“notch”) below the later plateau level of the AP and the initial notch was followed by a (very) early afterdepolarization (Figure 2). In some APs the notch was followed by a peculiar oscillation during plateau phase of the AP. This peculiarity of notch/oscillation was only observed in some of independent differentiation batches of the C25 cell line, but never in any other of three in-house cell lines or five commercial cell lines investigated with sharp microelectrodes as previously described [5,9,27,28,29,30]. When the notch/oscillation was detected in one EHT, all impalements showed this peculiarity including all EHTs from this differentiation batch. From all C25 batches investigated with sharp microelectrode, we detected seven with notch/oscillations and 11 without. The passage number of individual differentiation batches was not significantly different with or without notch/oscillations (77.1 ± 5.9, *n* = 7 vs. 67.9 ± 7.9, *n* = 9; *p* = 0.391) as well as the hiPSC-CM differentiation efficiency (81% ± 5% vs. 85% ± 3% TnT + cells, *p* = 0.51). In addition, spontaneous beating frequency was not different between EHTs with or without notch/oscillations (1.09 ± 0.19 Hz, *n* = 9 vs. 0.92 ± 0.07 Hz; *n* = 14; *p* = 0.34). EHTs were treated with 300 nM ivabradine to allow pacing at 1 Hz as previously described [5]. Under these conditions, APD_90_ was slightly longer in EHTs with notch/oscillations than without (279 ± 12 ms, *n* = 18/9/6 vs. 226 ± 6 ms, *n* = 30/17/9; *p* = 0.0044). Take-off potential, upstroke velocity and AP amplitude were not significantly different. The number of oscillations within one AP varied between 1 and 6. In case of several oscillations, there was a rather constant cycle length of oscillations in different EHTs (22.8 ± 0.9 ms, *n* = 11). In case of multiple oscillations, the amplitude decreased with each subsequent oscillation (ranging from 50 to 10 mV, Figure 2B). We used the specific inhibitor IBTX (100 nM) in order to evaluate whether oscillations originate from I_BK,Ca_. To quantify effects of IBTX on early repolarization we analyzed the membrane potential when the initial repolarization reached its lowest point (on average 9.2 ± 0.7 ms after AP upstroke).

**Figure 3:**
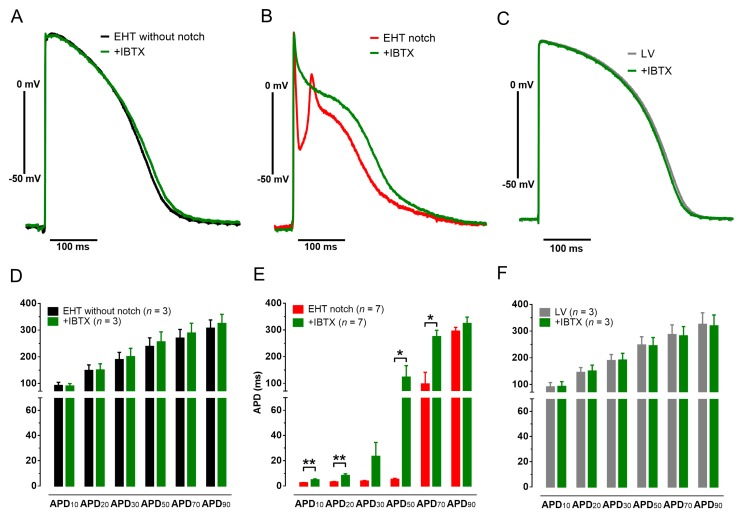
Effects of iberiotoxin (IBTX) on action potentials (APs) in C25 engineered heart tissue (EHT) and in human left ventricular tissue (LV). Original AP recordings before and after exposure to IBTX (100 nM, green) in EHTs with (**A**) and without (**B**) notch/oscillations from cell line C25 and in LV (**C**). Mean values of AP duration at different levels of repolarization (APD_10-90_) before and after exposure to IBTX (100 nM) in EHTs with (**D**) and without (**E**) notch in cell line C25 and in LV (**F**). Mean values ± SEM, * *p* < 0.05, ** *p* < 0.01, paired Student’s *t* test.

In case of notch/oscillations, IBTX (100 nM) lifted the membrane potential at this time point from −43.5 ± 5.6 to −13.2 ± 8.3 mV (*n* = 7, *p* = 0.001, paired *t*-test). APD_10_, APD_20_, APD_50_ and APD_70_ but not APD_90_ was significantly prolonged by IBTX (Figure 3). IBTX did not affect AP duration in EHTs without notch/oscillations. In addition, IBTX did not show any effect on AP in human LV tissue (Figure 3).

### 3.3. EHT with Notch/Oscillations in the AP Showed Large BK_Ca_ Expression

**Figure 4:**
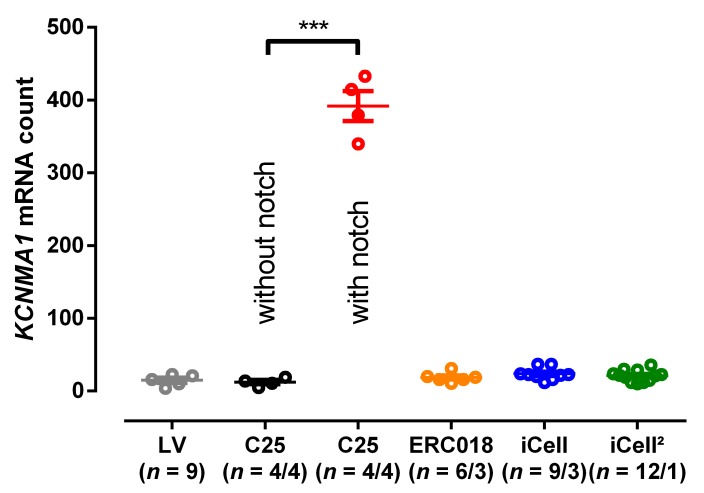
Expression analysis for the alpha subunit (*KCNMA1)* of big conductance calcium activated potassium channel. Individual expression levels of *KCNMA1* in left ventricular tissue (LV) and engineered heart tissue (EHT) from cell line C25 (without and with notch/oscillations), ERC018, iCell and iCell². Mean values ± SEM, *** *p* < 0.001, 1-way ANOVA with multiple comparison, *n* = number of patients for LV and *n* = number of EHT/number of individual differentiation batches.

The BK_Ca_ channel is formed as a tetramer of the channel forming alpha-subunit K_Ca_1.1 encoded by the *KCNMA1* gene. We performed retrospectively expression analysis in order to investigate whether there was an association between appearance of oscillations and the expression level of *KCNMA1*. The mRNA level for the channel forming alpha-subunit K_Ca_1.1 showed very low expression in human LV tissue. The same holds true for the in-house control cell line ERC018 and the commercially available cell lines iCell and iCell², where notch or oscillations in the AP have never been detected [5]. In contrast, all EHTs from C25 from which we could record notch/oscillations showed a substantial increase in *KCNMA1* mRNA, which is encoding for K_Ca_1.1 (Figure 4). In addition, RNA sequencing showed a 12-fold higher expression level for *KCNMA1* in C25 than in the control cell line ERC001 (Supplementary Figure S4). Other genes related to *KCNMA1* did not show major alterations in expression. Immunofluorescence analysis of hiPSC-CMs revealed the expression of the alpha-1 subunit of the BK_Ca_ channel in the C25 cell line with enhanced signal intensity at cell-cell contacts, whereas no signal was detected in the control cell line [30] (Supplementary Figure S5).

### 3.4. Computer Simulations Corroborate the Effect of the Non-Cardiac BK_Ca_ to Human AP

**Figure 5:**
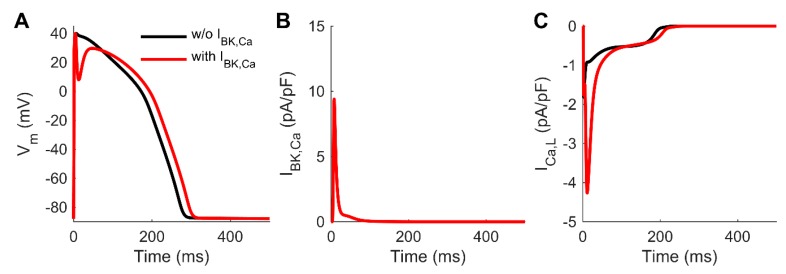
Impact of including BK_Ca_ in a mathematical model of human ventricular cardiomyocytes. BK_Ca_ current (**B**) causes a deep notch in the action potential (**A**), and a substantial increase in the amplitude of L-type Ca^2+^ current (**C**).

To evaluate the impact of BK_Ca_ on repolarization quantitatively, we integrated a BK_Ca_ model into a human ventricular CM model. Simulation results in Figure 5 demonstrated that a reasonably sized BK_Ca_ current can cause a deep notch in the AP. The dynamics of the notch (Figure 5A) match the kinetics of the simulated BK_Ca_ current (Figure 5B). Although inclusion of BK_Ca_ did not cause oscillations of the membrane voltage similar to in vitro observations, the deep notch enhanced drastically L-type Ca^2+^ current (I_Ca,L_, Figure 5C).

## 4. Discussion

The main findings of this study were:

(1) HiPSC-CMs could express ion channels otherwise not found in adult cardiomyocytes.
(2) In human adult ventricular myocardium, BK_Ca_ did not contribute to AP shape.
(3) In hiPSC-CMs BK_Ca_ could induce irregular AP shapes with oscillations resembling very early after depolarizations.

BK_Ca_ channels are widely expressed in various cell types, including electrically excitable and non-excitable cells [31]. Due to the Ca^2+^-sensitivity, they provide relevant negative feedback mechanism in the regulation of intracellular Ca^2+^ elevation and membrane potential [32]. Almost every cell type expresses BK_Ca_ in the inner mitochondrial membrane (BK_mito_) [33]. In CMs, pharmacological opening of BK_mito_ reduces ischemia reperfusion injury [34]. Sarcolemmal expression of BK_Ca_ is typically found in vascular smooth muscle cells, regulating myogenic tone and thereby blood flow. In CMs from rodents and cardiac tissue from humans, sarcolemmal expression of BK_Ca_ is very low or almost non-existent [35,36,37]. Here we confirm low expression levels of BK_Ca_ in human LV myocardium. Among CMs from other species, IBTX sensitive currents were found only in cultured embryonal chicken CMs [38]. Contribution to repolarization in this species is unclear. In rat ventricular myocardium, IBTX does not affect AP shape [39]. Here, we demonstrated for the first time that human ventricular APs were insensitive to IBTX. In consequence, the detection of I_BK,Ca_ in C25-hiPSC-CMs during patch clamp recordings was unexpected and surprising, since BK_Ca_ is not known to be expressed in the sarcolemma. The effectivity of the selective blocker of I_BK,Ca_ [26,40], IBTX, which only binds from the external side of the channel [41], confirmed the hypothesis that a non-cardiac channel is present and active at transmembrane level in the hiPSC-CMs. These findings were also confirmed by expression analysis in which the existence of the notch/oscillation in the AP correlated with the high expression levels of *KCNMA1*, encoding for the alpha-subunit (K_Ca_1.1) of the BK_Ca_. In contrast, *KCNMA1* expression was low in human LV tissue and correspondingly IBTX did not show any effect on APs from human LV, which to our knowledge has not been described before.

Recently, overexpression of non-cardiac BK_Ca_ in CMs was proposed as a treatment for LQTS [13], since BK_Ca_ is a hyperpolarizing channel, which might shorten human AP. Support for this idea came from a study describing the electrophysiological function of BK_Ca_ in HL-1 cells by viral overexpression, a cell line derived from a murine atrial tumor. BK_Ca_ overexpression reduced the very short AP of this murine model by 50% (APD_90_ from 30 to 14 ms) and was proposed as a potential genetic therapy to reduce AP duration (APD) of the LQT syndrome [13]. In contrast to the experiments in HL-1 cells, we observed that in hiPSC-CMs, the presence of BK_Ca_ induces oscillations in the early plateau phase and no speed-up in the final repolarization [13].

The pronounced initial repolarization could be confirmed by the in-silico integration of the I_BK,Ca_ in modeling ventricular myocyte AP (Figure 5). However, the inclusion of BK_Ca_ could not resemble oscillations of the membrane voltage. Nevertheless, we would expect that oscillation might be induced by an alternating feedback mechanism [32] of the I_BK,Ca_ and the L-type Ca^2+^ current (I_Ca,L_), since the deep notch drastically enhanced L-type Ca^2+^ current (Figure 5C). The afterdepolarizations following the initial repolarization might be also due to activation of I_Ca,T_ [11] or the sodium calcium exchanger, however, the exact mechanism remains unclear. More accurate and detailed mathematical modeling would require in vitro data on spatial distribution and localization of the BK_Ca_ and Ca^2+^ channels, which is beyond the scope of this study.

Afterdepolarizations might complicate the evaluation of how the BK_Ca_ affects APD. In vitro, there was a slightly longer APD_90_ when BK_Ca_ was present, which could be confirmed in silico. However, the inhibition of I_BK,Ca_ by iberiotoxin in AP with notch/oscillations did not significantly alter APD_90_, averaged values tended even to longer APD_90_. The apparent differential contribution of BK_Ca_ to the APD revealed at baseline level or by pharmacological intervention might be due to remodeling of other ion channels downstream to BK expression or due to potential off-target effects of iberiotoxin. Taken together, our results do not support the idea that BK_Ca_ overexpression can cure LQTS in humans, since BK_Ca_ might lead to afterdepolarization and arrhythmia.

Previously, it was shown that the I_BK,Ca_ current contribute to outward currents in the murine sinoatrial node and the selective blocker paxilline decreased beating rate by more than 50% [42]. However, the exact role of I_BK,Ca_ in pacemaking is widely unclear, since substantial decrease in diastolic depolarization by paxilline does not fit to very small contribution of BK_Ca_ to total potassium outward currents activated at positive membrane potentials. Nevertheless, spontaneous beating is a peculiarity of hiPSC-CMs and the autonomic activity of hiPSC-CMs is not fully understood. Therefore, it seems reasonable to speculate that BK_Ca_ may be involved in pacemaking of hiPSC-CMs. However, we would not expect a large impact of I_BK,Ca_ to pacemaking in EHT, since beating rate in EHT with and without expressing BK_Ca_ did not differ.

The reason for the unexpected expression of BK_Ca_ in individual differentiation batches of a single hiPSC-CM cell line (C25) is very difficult to evaluate retrospectively. Cell line C25 did not show chromosomal anomalies at passage number 40 and 92 (Supplementary Figure S3). Since 6 out of 7 individual differentiation batches were done from lower passage numbers than 92, it seems that BK expression was not the consequence of karyotype abnormalities. As notch/oscillation were observed also in preparations with very high differentiation efficiencies (90% and 93%) and hiPSC-CM fraction in EHT was even enriched in comparison to 2D culture (Supplementary Materials Figure S2B), we are confident that BK expression is not due to an extraordinary high fraction of non-cardiac cells within the EHT. Strong batch effects, as either all or none of the EHTs from one preparation depicted signs of BK_Ca_ expression, argue for an upregulation of BK_Ca_ due to events occurring during stem cell culture and cardiac differentiation. There are two factors that might have raised the likelihood for spontaneous mutations in the stem-cell culture. The C25 cell line was reprogrammed by lentivirus and it was passaged to very high number (up to 107 passages). To avoid these factors, we changed to Sendai virus and restricted passage number for future experiments. Since *KCNMA1* related genes did not show major alterations in contrast to *KCNMA1* itself, we would account a genetic alteration more likely than an upregulation due to regulatory pathways. In addition, various reports show that iPS-cells frequently acquire genetic alterations in cell culture. Kilpinen et al. [43] showed that chromosome 10, harboring BK_Ca_/*KCNMA1*, was among the most susceptible loci to copy number alterations. In addition, in a study searching for variants that provide mutated cells with a growth advantage in culture, *KCNMA1* candidate mosaic variants were identified in two independent hES cell lines [44]. Thus, a genetic alteration leading to a reoccurring overgrowth of BK misexpressing hiPSC is the most likely explanation for our finding. Regulatory expression profiling might reveal this change of BK_Ca_ activity in advance.

## 5. Conclusions

Our results clearly demonstrated that hiPSC-CMs could possess even non-cardiac ion channels affecting AP waveform causing afterdepolarizations and oscillations in the AP. This might serve as an example that iPS cell culture could lead to genetic alterations with functional consequences. Therefore, we felt that cell culture and differentiation protocols should be standardized as much as possible and that expression of non-cardiac sarcolemmal ion channels should be considered before hiPSC-CMs are used for pharmacological studies. Screening for the expression of the *KCNMA1* gene might be one potential quality parameter.

## 6. Limitations

We are aware that the description of the BK_Ca_ in a single control cell line of hiPSC-CMs is of limited transferability, especially since we could not uncover conditions or mechanisms of the unexpected BK_Ca_ expression. Nevertheless, presence of BK_Ca_ is a good example that hiPSC-CMs can express non-cardiac proteins with a huge impact on physiological parameters. Although IBTX has been described as selective for I_BK,Ca_ [41], non-specific effects of IBTX cannot be completely excluded. LV tissue was obtained from patients with heart disease; a potential difference to healthy LV tissue is unclear. We implemented the BK in the previously developed hiPSC-CM model [5], but we failed to induce notch/oscillations in that model. Obviously, our present model does not reflect the cellular ultrastructure at the level of detail that would be required for simulating the putative close proximity of the BK and CaL channels.

## Supplementary Materials

The following are available online at https://www.mdpi.com/2073-4409/9/1/253/s1; Figure S1: Outward currents in iCell human induced pluripotent stem cell-derived cardiomyocytes (hiPSC-CMs) and the effect of iberiotoxin (IBTX) and Figure S2: Cardiomyocyte content analyzed by flow cytometry. Figure S3: Karyotype of hiPS-cells. Figure S4: RNA sequencing of BK related genes in hiPSC-CMs. Figure S5: Immunoflurorescence analysis of BK in hiPSC-CMs.

## Abbreviations

AP	Action potential
APD	Action potential duration
APD_90_	Action potential duration at 90% repolarization
BK_Ca_	Big conductance calcium activated potassium channel, (Maxi-K, slo1, K_Ca1.1_)
CM	Cardiomyocytes
EAD	Early afterdepolarization
EHT	Engineered heart tissue
hiPSC-CMs	Human induced pluripotent stem cell-derived cardiomyocytes
IBTX	Iberiotoxin
I_BK,Ca_	Big-conductance calcium activated potassium current
I_Ca,L_	L-type calcium current
LV	Left ventricle
RMP	Resting membrane potential
