# Supplementary material for: Case Report on: Very Early Afterdepolarizations in HiPSC-Cardiomyocytes—An Artifact by Big Conductance Calcium Activated Potassium Current (Ibk,Ca)
Source: Cells. 2020 Jan 20;9(1):253. doi: 10.3390/cells9010253 (PMC7017352; doi:10.3390/cells9010253)
Supplement: Supplementary file 1 [file cells-09-00253-s001.pdf]

<u>**Supplementary Figures**</u>

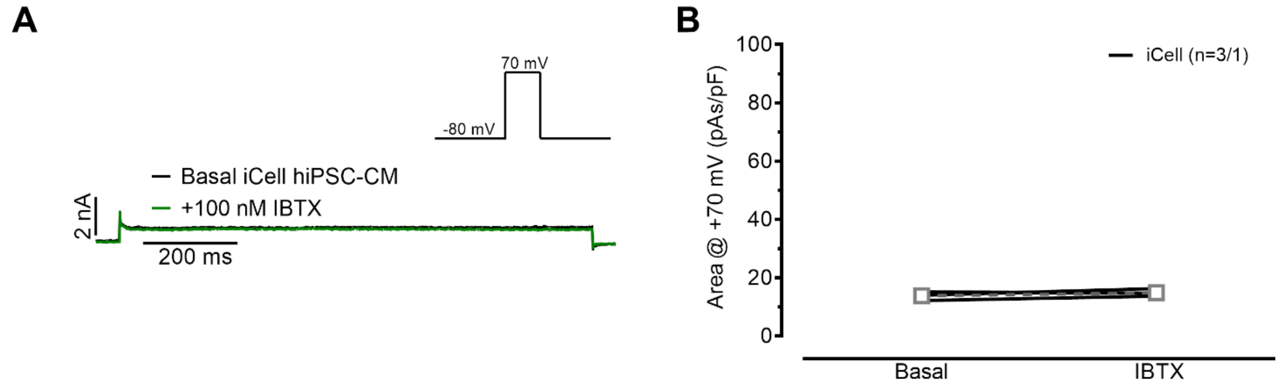

Supplementary Figure S1: Outward currents in iCell human induced pluripotent stem cell-derived cardiomyocytes (hiPSC-CMs) and the effect of iberiotoxin (IBTX). (A) Original outward traces before (black) and after (green) exposure of 100 nM IBTX in iCell hiPSC-CMs (upper, black directly underlying green curve). (B) Summary of IBTX (100 nM) effects in iCell hiPSC-CMs quantified by area under the curve. Mean values±SEM; non-significant in paired Student‘s *t*-test for basal vs. IBTX; n=number of isolated cells/number of individual differentiation batches. Both figures are adapted to the range of Figure 1 for better comparability.

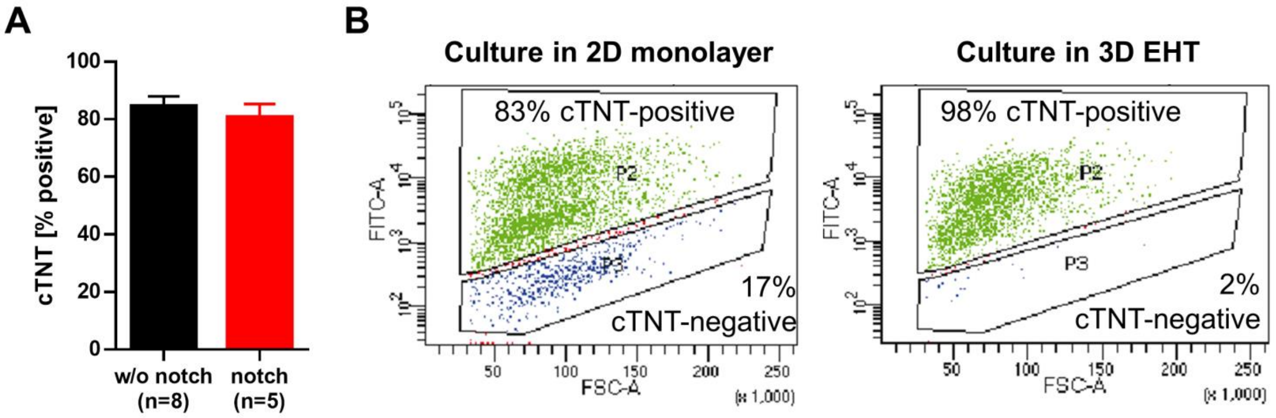

Supplementary Figure S2: Cardiomyocyte content analyzed by flow cytometry. (A) Yield of hiPSC-CMs after cardiac differentiation of the C25 line before casting into engineered heart tissues (EHTs), comparison of preparations resulting in EHTs without (w/o) and with notch. n=number of individual differentiation batches. Mean values±SEM; non-significant in paired Student‘s *t*-test. (B) Comparison of cardiac Troponin T (cTNT) positive cells (green) by flow cytometry after culturing C25 hiPSC-CMs for 6 weeks in 2D monolayer (left, 83%) or 3D EHT (right, 98%). Culture in 2D and 3D was performed in parallel from the same batch of C25 hiPSC-CMs.

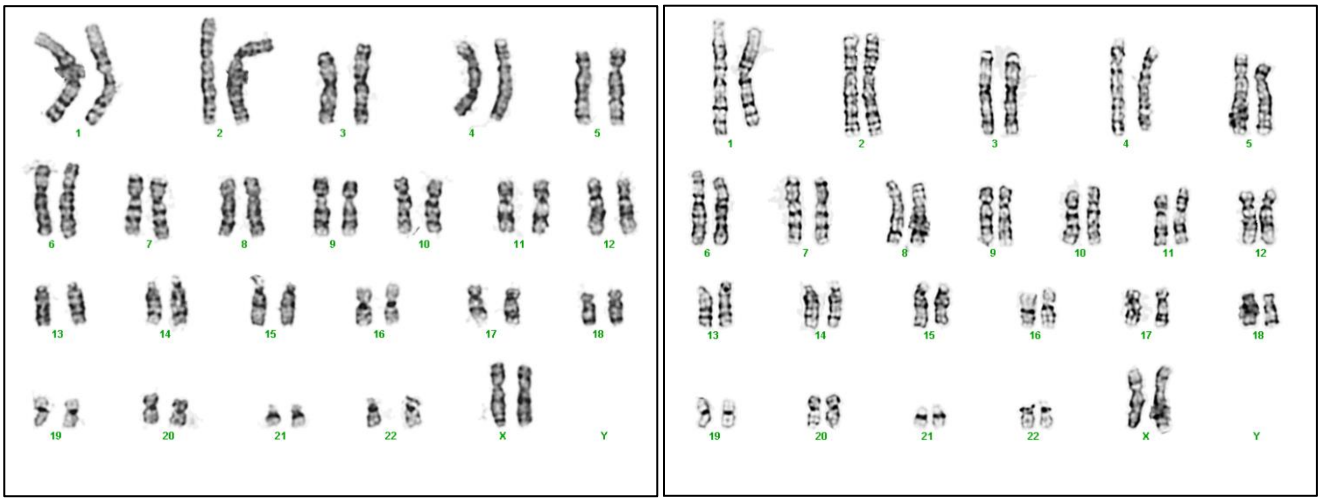

Supplementay Figure S3. Karyotype of hiPS-cells. The karyotype of C25 iPS-cells were investigated at passage number 40 (left) and 92 (right) without showing chromosomal anomalies.

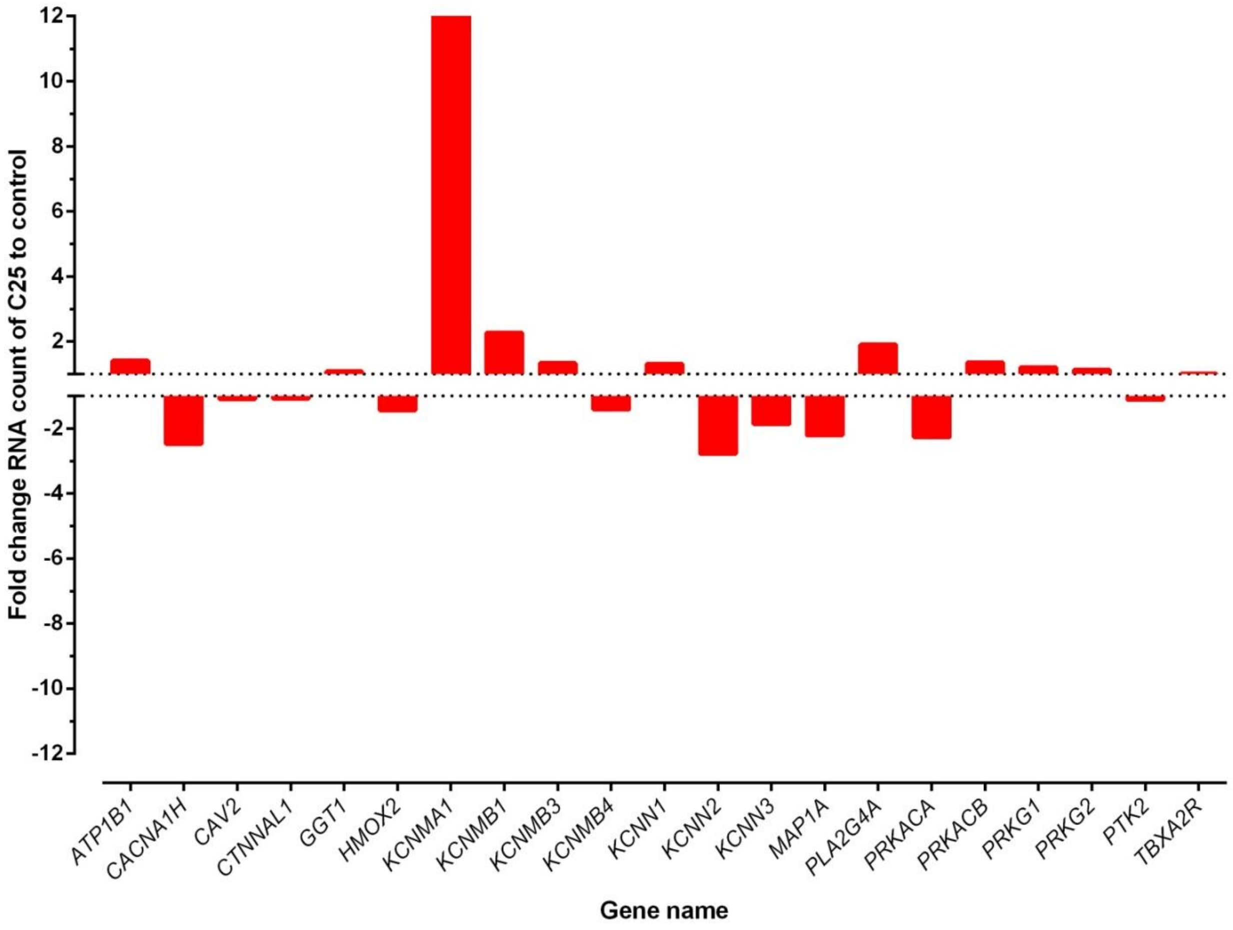

Supplementary Figure S4. **RNA sequencing of BK related genes in hiPSC-CM.** RNA sequencing revealed 12 fold higher expression levels for *KCNMA1* in C25 than in the control cell line ERC001. Other genes related to *KCNMA1* (discovered by https://version11.string-db.org/cgi/network.pl?taskId=orTwBJuZdhk3) did not show major alterations in expression. *AC117457.1, KCNMB2, KCNN4, KCNU1, PRKACG* were not detected in both cell lines.

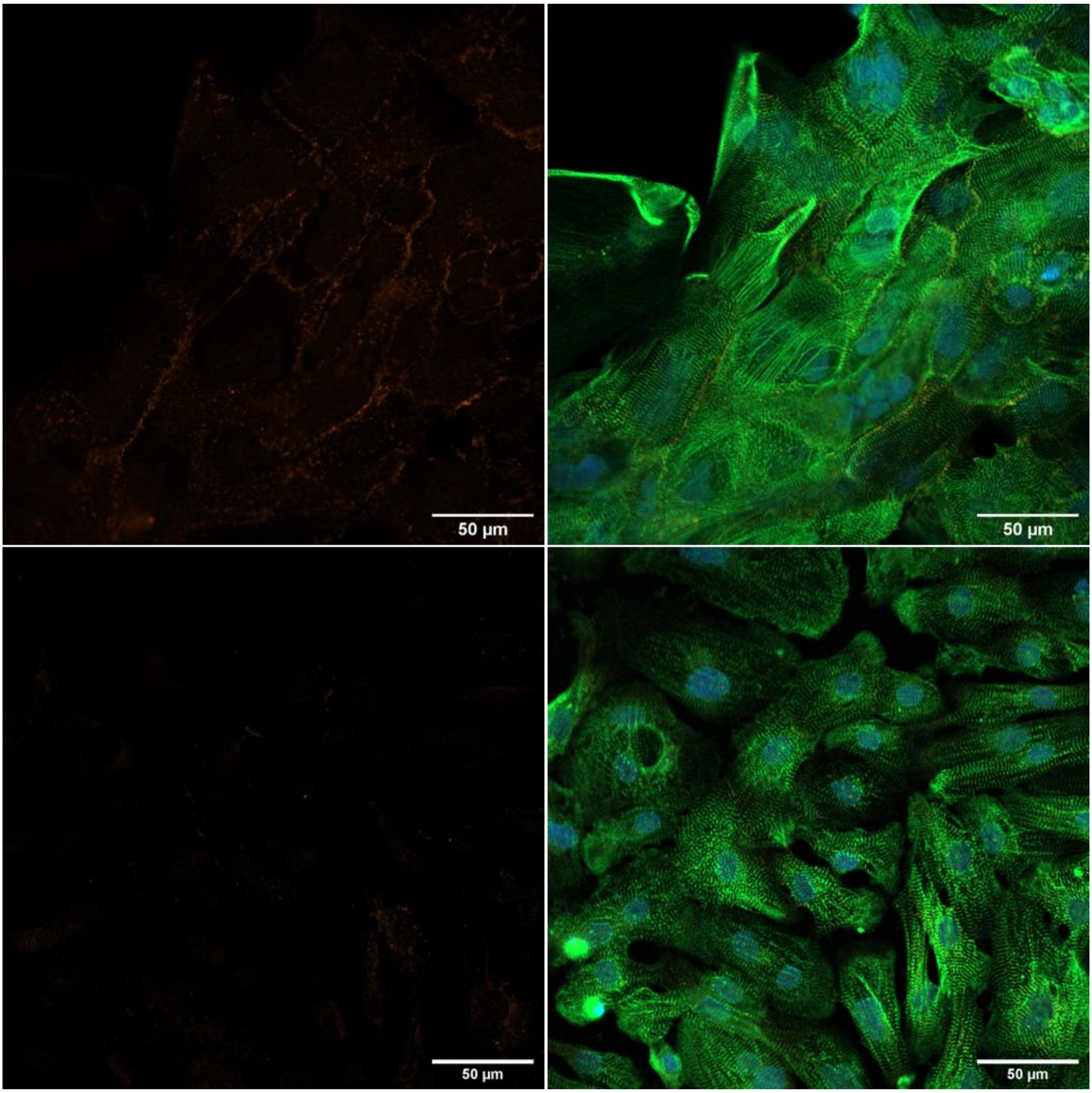

Supplementary Figure S5. Immunofluorescence analysis of BK in hiPSC-CMs. Subcellular localization of the large conductance calcium-activated potassium channel (subfamily M subunit alpha-1; BK, antibody: Alomone extracellular antibody APC-151, orange, left side) and merged image (right side of α-actinin (green) and nuclear staining (blue) in a 2D culture of hiPSC-CMs from the C25 (upper panel) and an independent control cell line (lower panel).

Supplementary Methods

RNA isolation and gene expression analysis

For RNA sequencing, RNA integrity was analyzed with the RNA 6000 Nano Chip on an Agilent 2100 Bioanalyzer (Agilent Technologies). mRNA was extracted from total RNA using the NEBNext Poly(A) mRNA Magnetic Isolation module (New England Biolabs) and RNA-Seq libraries were generated using the NEXTFLEX Rapid Directional qRNA-Seq Kit (Bioo Scientific) as per the manufacturer´s recommendations. Concentrations of all samples were measured with a Qubit 2.0 Fluorometer (Thermo Fisher Scientific) and fragment lengths distribution of the final libraries was analyzed with the DNA High Sensitivity Chip on an Agilent 2100 Bioanalyzer (Agilent Technologies). All samples were normalized to 2 nM and pooled equimolar. The library pool was sequenced on the NextSeq500 (Illumina) with 1x75bp, with 16.6 to 21.7 mio. reads per sample. For each sample the quality of the raw reads was confirmed by FastQC v0.11.8 (http://www.bioinformatics.babraham.ac.uk/projects/fastqc). The reads were aligned to the human reference genome GRCh38 with STAR v2.7.0f (Dobin et al, 2013) and simultaneously counted per gene by employing the *quantmode GeneCounts* option. Counts are based on the Ensembl annotation release 96. Normalization factors for each sample were estimated with *DESeq2* v1.24.0 (Love et al, 2014) and used to generate normalized coverage tracks with *bamCoverage* from *deepTools* v3.2.1 (Ramirez et al, 2016).

Immunofluorescence staining of hiPSC-derived cardiomyocytes

HiPSC-CMs were cultured for 30 days in 96-well plates (µclear®, Greiner) and subsequently prepared for immunofluorescent analysis, as described previously (Prondzynski et al, 2017). Staining for the alpha-1 of the BK_{Ca} channel was performed by a primary antibody (1:200, APC-151 Alomone) and a secondary goat anti-rabbit Alexa Fluor® 546 antibody (1:800, A11035 Life Technologies). Staining for α-actinin 2 was performed by a primary antibody (1:800, A7811 Sigma) and a secondary goat anti-mouse Alexa Fluor® 488 antibody (1:800, A11029 Life Technologies).The nucleus was stained with Hoechst 33342 (1:2,500, Thermo Fischer Scientific) and images were obtained by confocal microscopy using the Zeiss LSM 800 confocal microscope on the same day with the same laser power and detector setting.
